# Initial Evaluation of a Novel Cone-Beam CT-Based Semi-Automated Online Adaptive Radiotherapy System for Head and Neck Cancer Treatment – A Timing and Automation Quality Study

**DOI:** 10.7759/cureus.9660

**Published:** 2020-08-11

**Authors:** Suk Whan Yoon, Hui Lin, Michelle Alonso-Basanta, Nate Anderson, Ontida Apinorasethkul, Karima Cooper, Lei Dong, Brian Kempsey, Jaclyn Marcel, James Metz, Ryan Scheuermann, Taoran Li

**Affiliations:** 1 Radiation Oncology, University of Pennsylvania, Philadelphia, USA; 2 Radiation Oncology, Perelman School of Medicine, University of Pennsylvania, Philadelphia, USA

**Keywords:** online adaptive radiotherapy, adaptive radiotherapy, image segmentation, head and neck oncology, automatic planning

## Abstract

Introduction

A novel on-line adaptive radiotherapy (ART) system based on O-ring linear accelerator (LINAC) and cone-beam CT (CBCT) was evaluated for treatment and management of head & neck (H&N) cancer in an emulated environment accessed via remote desktop connection. In this on-line ART system, organs-at-risk (OARs) and target contours and radiotherapy (RT) plans are semi-automatically generated based on the patient CBCT, expediting a typically hours-long RT planning session to under half an hour. In this paper, we describe our initial experiences with the system and explore optimization strategies to expedite the process further.

Methods

We retroactively studied five patients with head and neck cancers, treated 16-35 fractions to 50-70 Gys. For each patient, on-line ART was simulated with one planning CT and three daily CBCT images taken beginning, middle, and end of treatment (tx). Key OAR (mandible, parotids, and spinal cord) and target (planning target volume (PTV) = clinical target volume (CTV) + 3 mm margin) contours were auto-generated and adjusted as needed by therapist/dosimetrist and attending physician, respectively. Duration of OAR contouring, target contouring, and plan review was recorded. Key OAR auto-contours were qualitatively rated from 1 (unacceptable) - 5 (perfect OAR delineation), and then quantitatively compared to human-adjusted “ground truth” contours via dice similarity coefficient (DSC) and 95-percentile Hausdorff distance (HD95%). Once contours were approved, adapted RT plans were auto-generated for physician review. Simulated doses to OARs and targets from the adapted plan were compared to that from the original (un-adapted) plan.

Results

Median on-line ART planning duration in the remote emulated environment was 19 min 34 sec (range: 13 min 10 sec - 31 min 20 sec). Automated key OAR quality was satisfactory overall (98% scored ≥3; 82% ≥4), though mandible was rated lower than others (p < 0.05). Most key OARs and all targets were within 2 mm margin of human-adjusted contours, but a few parotid and spinal cord contours deviated up to 5 mm. Anatomical changes over tx course further increased auto-contour error (p < 0.05, ΔHD95% = 0.77 mm comparing start and end of tx). Further optimizing auto-contoured OAR and target quality could reduce the on-line treatment planning duration by ~5 min and ~4.5 min, respectively. Dosimetrically, adapted plan spared OARs at a rate much greater than random chance compared to the original plan (χ^2 ^= 22.3, p << 0.001), while maintaining similar therapeutic dose to treatment target CTV (χ^2 ^= 1.14, p > 0.05). In addition, a general decrease in accumulated OAR dose was observed with adaptation. Unsupervised adapted plans where contours were auto-generated without human review still spared OAR at a greater rate than the original plans, suggesting benefits of adaptation can be maintained even with some leniency in contour accuracy.

Conclusion

Feasibility of a novel, semi-automated on-line ART system for various head and neck (H&N) cancer sites was demonstrated in terms of treatment duration, dosimetric benefits, and automated contour accuracy in a remote emulator environment. Adaptive planning duration was clinically viable at 19 min and 34 sec, but further improvements in automated contour accuracy and performance improvements of plan auto-generation may reduce adaptive planning duration by up to 10 minutes.

## Introduction

Modern external beam radiation therapy (EBRT) for locally advanced head and neck (H&N) cancer aims to spare sensitive salivary glands and other functionally critical organs from radiation while improving loco-regional tumor control [1]. The advent of computerized dose optimization such as intensity modulated radiation therapy (IMRT) and image-guided radiation therapy (IGRT) substantially improved radiation dose conformity to primary tumors and high-risk nodes for H&N cancer, which theoretically mitigates negative side-effects to surrounding functional organs-at-risk (OAR) [2]. However, acute oral and pharyngeal side-effects due to radiation toxicity persisted with IMRT [3-7], because a combination of factors moved critical OARs into radiation fields during the treatment course including tumor response, inflammation, muscle atrophy, and patient weight [5, 8, 9]. Indeed, static IMRT field for a shifting patient anatomy results in a heightened radiation dose to these critical OARs that increases overall radiation toxicity [10].

In traditional radiotherapy (RT), static beams initially optimized based on a computed tomography (CT) scan is used throughout the treatment course; in comparison, adaptive radiotherapy (ART) radiation beams conform to the shifting patient anatomy. The success of ART depends on an accurate representation of the on-the-day patient anatomy to delineate the daily target and OAR volumes as real-to-life as possible [11]. CT-on-rails [12], on-board cone-beam CT (CBCT) [13], and magnetic resonance imaging (MRI) [14] have been used to capture daily anatomy, each modality with unique benefits and caveats ranging from superior soft-tissue imaging for MRI and superior radiological accuracy for CT, but CBCT is the most common since it comes integrated with modern medical linear accelerators (LINAC). However, CBCT suffers more from image artifacts and noise than CT, on top of longer acquisition time. Nonetheless, the feasibility of ART with CBCT is demonstrated in studies where planning CT is deformably registered to CBCT to generate a synthetic daily CT with which to re-plan [14, 15].

Daily adaptive re-planning strategies based on the daily CBCT images can be off-line or on-line, where the former retroactively plan RT on the previous day’s images and the latter plan RT on the day of image acquisition. Both off-line and on-line adaptive strategies based on CBCT images have produced clinically significant results for prostate cancer [13, 16]. For H&N cancer, off-line ART with one re-plan using a new diagnostic CT scan mid-course is now often practiced, with demonstrated improvement in loco-regional control and acceptable normal tissue toxicity [17]. However, implementing on-line H&N ART is technically challenging and impractical due to the sheer number of H&N OARs that need to be contoured on the spot as the patient awaits treatment. A practical solution for on-line H&N ART with CBCT would need to: (1) acquire images quickly, (2) aid rapid contouring of all relevant OARs and targets, and (3) aid rapid plan generation.

Hardware improvements in LINAC designs as well as software advances in contouring, deformation, and knowledge-based planning in recent years reduced the time-scale of on-line ART for H&N cancer from hours to minutes. Novel O-ring LINACs accelerated image acquisition from minutes to sub-minute and improved image quality via rapid iterative reconstruction algorithms [18]. Atlas-based auto-segmentation (ABAS) and CT-to-CBCT deformable propagation of OAR contours made the deformable transfer of original contours defined on the initial planning CT to daily CBCT rapid and practical [19]. Knowledge-based planning (KBP), an automated planning approach where a database of already-existing high-quality RT plan is queried for the closest match and deformed to the current geometry, significantly reduced planning duration [20, 21].

This paper describes initial experiences with an integrated on-line ART treatment planning and delivery system for H&N cancer, Ethos™ (Varian Medical Systems, Palo Alto, CA), which combines hardware capabilities of the novel O-ring LINAC with auto-contouring and auto-planning software capabilities. The system is semi-automated, in that human input drives automation and all final contour or treatment decisions are deferred to human review. Overall workflow and time expended in each step of the process are described in the context of clinical feasibility (i.e., expected to wait-time on the treatment couch) and potential for optimization is discussed. The quality of automated contours and RT plans is also evaluated and discussed.

## Materials and methods

Initial treatment planning

**Figure 1:**
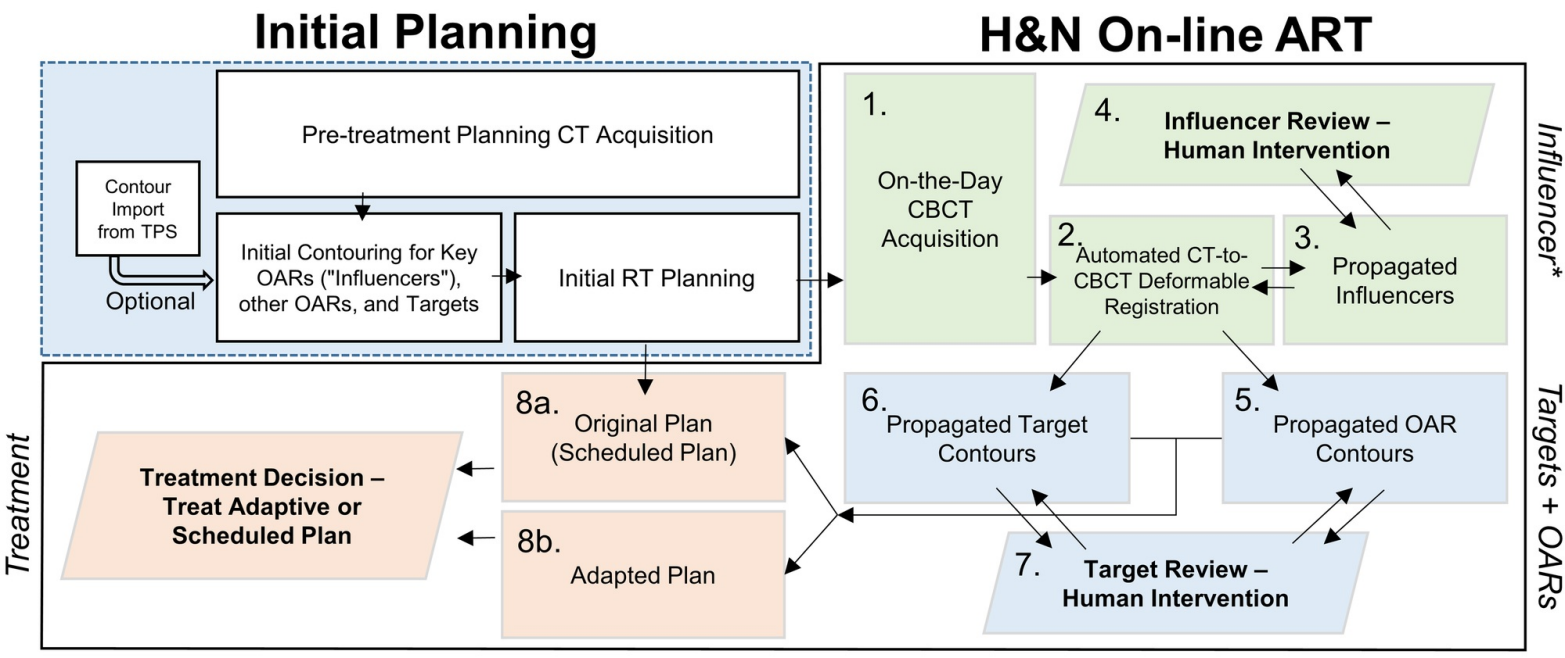
On-line ART workflow for H&N patients. During initial planning, pre-treatment planning CT is acquired and OAR and target contours are either imported from TPS or manually contoured within the adaptive planning software. On-the-day H&N on-line ART begins with 1. CBCT acquisition. 2. Planning CT is deformably registered to this CBCT (unique to H&N workflow). 3. Influencer is propagated (unique to H&N workflow). 4. Influencer is reviewed, from which an adjusted deformation field is derived. This human-adjusted deformation field is used to propagate. 5. OAR and 6. target contours which are also 7. reviewed. Corrected contours are used to plan and calculate dose from 8a. original (also called scheduled) and 8b. supervised adapted plans. *Influencers are OAR contours with clinical significance, which for H&N sites are the parotids, spinal cord, and mandible. Human modifications to these contours "influence" the CT-to-CBCT deformation vector field. ART: Adaptive radiotherapy; OAR: Organs-at-risk; TPS: Treatment planning software; H&N: Head & neck; CBCT: Cone-beam CT; RT: Radiotherapy.

Institutional ethical approval was obtained before the study was carried out under IRB #833974. The on-line ART process (Figure 1) starts with pre-treatment planning CT acquisition and initial treatment planning. In this step, predetermined sets of four key OARs or “influencers” (left parotid, right parotid, mandible, and spinal cord) and the rest of the OARs and treatment targets (low-, mid-, or high-risk clinical target volumes or CTV) are manually contoured by qualified operators or imported from other treatment planning software (TPS). Influencers are clinically significant contours that an operator can review during adaptation and alter as needed to guide the deformation vector field matching the planning CT to the CBCT. Planning risk volume (PRV) and planning treatment volume (PTV) margin expansions are specified in this step, which are adaptively applied to the deformably propagated on-the-day contours. In this paper, a PTV margin expansion of 3 mm is used throughout. Technical contours (such as titanium implants, artifacts in the CT) are auto-generated for operator review. Operators specify treatment planning parameters in the RT physician’s intent module, including dose per fraction, clinical goals (e.g., unilateral parotid mean dose, D_mean_, must be <26 Gy; or least dose to most-irradiated 0.03 cm^3^, D 0.03 cm^3^, of spinal cord must be <50 Gy), and treatment technique (number of IMRT beams, IMRT vs. VMAT). Unlike in traditional RT planning, the system automatically determines beam angles and isocenter placements from its Intelligent Optimization Engine, which is a plan optimization system based on clinical goal priorities set by the operator. These priorities allow the operator to prioritize clinical goals for critical OARs if their volumes are within a dosimetric falloff margin of the treatment target, or vice versa if treatment target is to be prioritized over the OAR. A list of clinical goals and user-determined priorities are provided (Table 1). A dose preview with a list of clinical goals expected to be met based on current patient geometry is generated for physician approval or replanning, if unsatisfactory.

**Table 1:** Clinical goals and corresponding priorities used for initial planning. *One (1) patient had a single PTV prescribed to 50 Gy. **A range of dose limits were used for parotids and submandibular glands depending on the location of PTV. PTV: Planning target volume; PRV: Planning risk volume.

Structure Name	Clinical Goal	Variation Acceptable	Priority
Spinal Cord	D0.03cc[Gy]<=45	45	1
Spinal Cord + 5 cm PRV	D0.03cc[Gy]<=50	50	1
Brain Stem	D0.03cc[Gy]<=54	54	1
Left Eye	D0.03cc[Gy]<=45	45	1
Right Eye	D0.03cc[Gy]<=45	45	1
Optic Chiasm	D0.03cc[Gy]<=54	54	1
Left Optic Nerve	D0.03cc[Gy]<=54	54	1
Right Optic Nerve	D0.03cc[Gy]<=54	54	1
PTV_High	D95%[Gy]>=70(50)*	70(45)*	2
PTV_High	D99%[Gy]>=65(46)*	65(45)*	2
PTV_Mid	D95%[Gy]>=63	63	2
PTV_Mid	D99%[Gy]>=59	59	2
PTV_Low	D95%[Gy]>=56	56	2
PTV_Low	D99%[Gy]>=52	52	2
Mandible - PTV	D0.03cc[Gy]<=70	70	2
Left Parotid	Mean[Gy]<=16-26	16-26**	2
Right Parotid	Mean[Gy]<=16-26	16-26**	2
Left Cochlea	Mean[Gy]<=30	30	2
Right Cochlea	Mean[Gy]<=30	30	2
Esophagus - PTV	Mean[Gy]<=20	20	2
Left Submandibular Gland	Mean[Gy]<=30-36	30-39**	2
Right Submandibular Gland	Mean[Gy]<=30-36	30-39**	2
Larynx - PTV	Mean[Gy]<=20	20	2
Left Temporal Lobe	Mean[Gy]<=25	25	2
Right Temporal Lobe	Mean[Gy]<=25	25	2
Pharynx Constrictor	Mean[Gy]<=50	50	2
Pharynx Constrictor - PTV	Mean[Gy]<=40	40	2
Oral Cavity - PTV	Mean[Gy]<=20	20	2
External / BODY	D0.03cc[Gy]<=77	77	2

Head & neck online adaptive treatment planning

Adaptive planning and delivery can be subdivided into three modules: (A) Influencer contour generation (steps 1-4 on Figure 1), (B) target contour generation (5-7), and (C) adapted plan generation and treatment decision (8a,b). Influencer and target contours can be revised to correct imperfections in the auto-contouring processes in steps 4 and 7 if needed. During module (A), planning CT is deformably registered to the acquired on-the-day CBCT to propagate auto-generated influencers (from the planning stage) to the on-the-day CBCT. The operator may intervene if the propagated influencers are unsatisfactory. The resulting human-adjusted influencers are used to "influence” or correct the deformation field, which is in turn used in module (B) to propagate other OARs and targets (i.e., CTV). If the resulting target and OAR contours are unsatisfactory, the operator can once again intervene to correct for any defects. All contours are then fed into the original RT plan and re-optimized for module with identical optimization constraints as the original plan (C). During this final step, calculated doses and dose-volumes for each contour along with respective clinical goals are displayed for both the adapted plan and the original plan. If the adapted plan is unsatisfactory, the physician may decide to treat with the original plan.

Auto-contour quality assessment

The on-line ART processes described above were used to retroactively study five patients with head and neck cancers treated with daily CBCT guidance on an O-ring LINAC, on an emulated environment accessed via remote desktop connection. For each patient, on-line ART was simulated twice, supervised (where influencer and target contours were reviewed by delineators) and unsupervised (without human review), with one planning CT and three daily CBCT images taken beginning, middle, and end of treatment for 15 x 2 on-line sessions total. Key influencer OAR and target (CTV, with adaptive margin + 2 mm for PTV generation) contours were auto-generated on both runs, but adjusted by therapist/dosimetrist and attending physician respectively on the supervised run. Time expenditure in each step of the process during the supervised run was recorded for each of the 15 sessions. Auto-contours were qualitatively rated by consensus between the delineator and the physician from 1 (unacceptable) to 5 (perfect OAR delineation), and then quantitatively compared to human-adjusted contours via dice similarity coefficient (DSC) and 95-percentile Hausdorff distance (HD95%). All statistical tests were carried out on Microsoft Excel (Microsoft, Redmond, WA).

Adaptive plan quality assessment

All OAR and target dose constraints calculated in this paper use the supervised human-adjusted contours as defined in the Auto-Contour Quality Assessment subsection, since these contours most closely represent ground truth daily contours. For example, “mean dose (D_mean_) to parotids from the original un-adapted (scheduled) plan” refer to the mean dose to human-adjusted parotid contours, not planning contours. Daily adapted and original un-adapted plans were compared in terms of the number of clinical goals / dose constraints met for human-adjusted OAR and target contours. Accumulated dose for one patient treated with un-adapted plan was compared to that from supervised adapted plan. The treatment plan auto-generated based on unsupervised contours was compared with the original un-adapted plan in terms of their similarity to the supervised adapted plan.

## Results

Timing data and operator decision statistics

**Figure 2:**
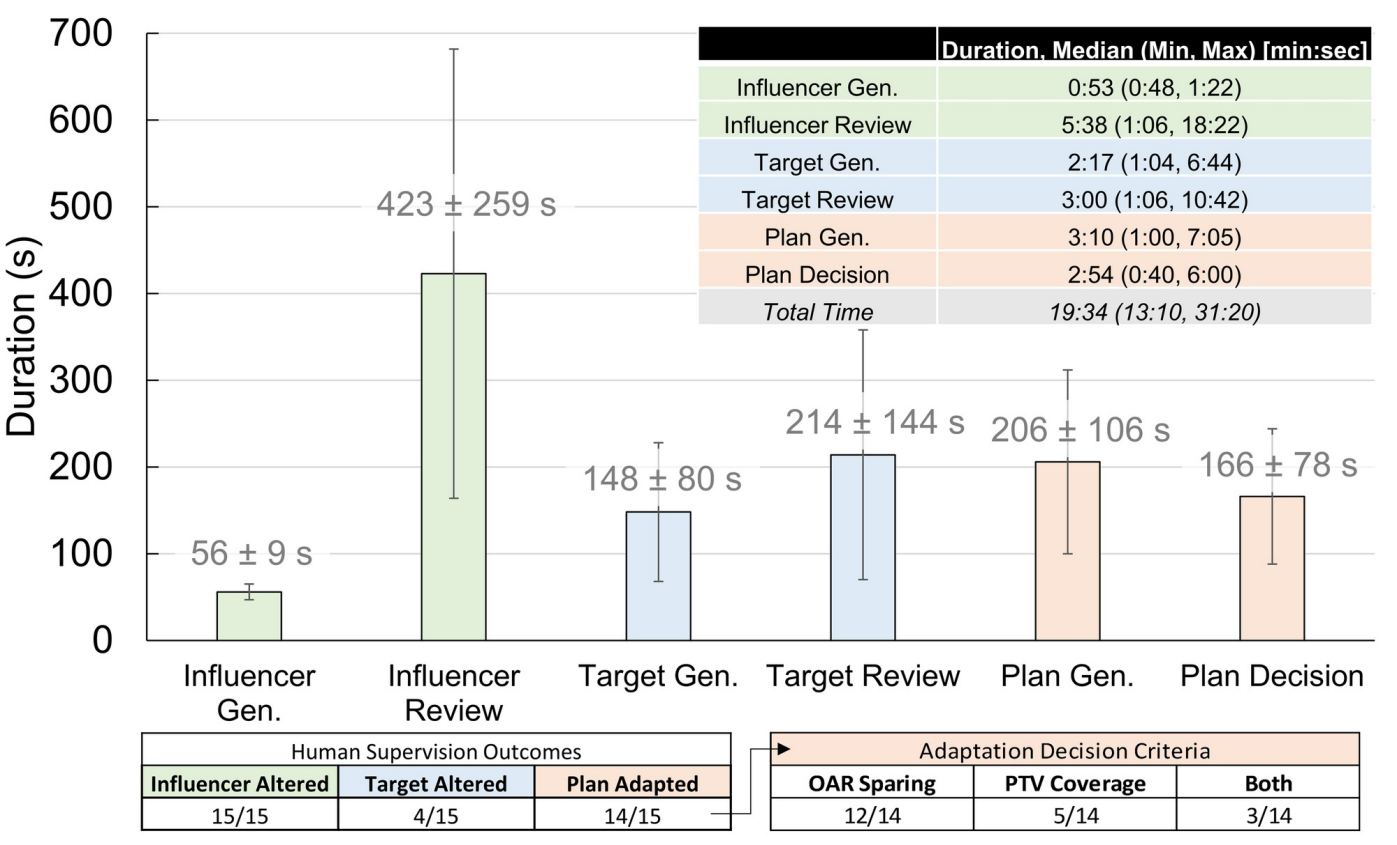
Overview of on-line ART workflow duration in each module and summary of human intervention outcomes. ART: Adaptive radiotherapy; OAR: Organs-at-risk; PTV: Planning target volume.

Figure 2 summarizes descriptive statistics of timing and human review outcomes on an emulated on-line ART system accessed remotely. Median time spent for on-line ART, not including patient set-up, image acquisition, and treatment delivery, was 19:34 (min:sec), with a range of 13:10 to 31:20. Automated influencer, target, and plan generation took a median of 0:53, 2:17, and 3:10, respectively. All automated influencers required human adjustment, while targets generated after human adjustments to the influencers were in general satisfactory, with 4/15 automated targets requiring contour revision. Adapted plan after target review was favored over original plan for treatment for 14/15 supervised sessions mostly due to superior OAR sparing (12/14), sometimes superior PTV coverage (5/14), or both (3/14). One case was not adapted because the physician observed little difference in terms of clinical goals between the adapted plan and the original (scheduled) plan.

Contour accuracy

**Figure 3:**
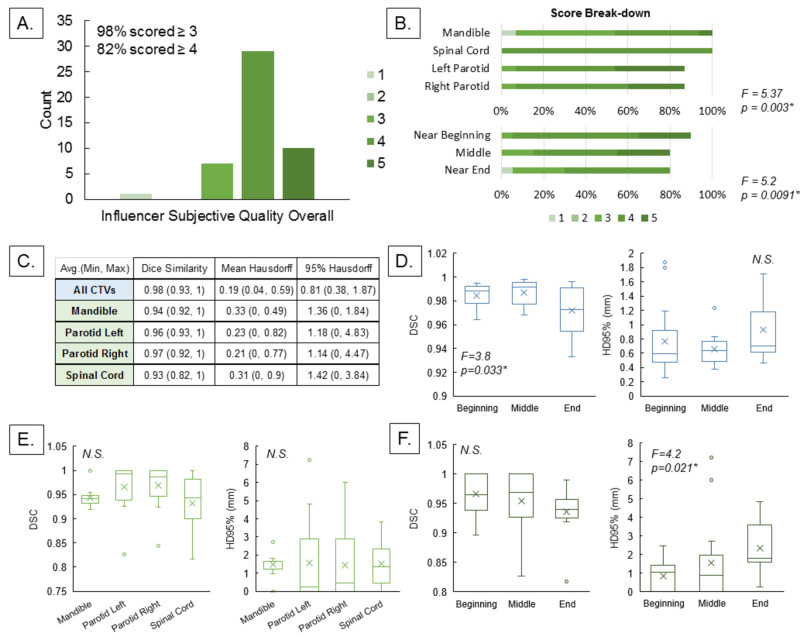
Subjective and objective comparisons of auto-generated influencer and target contours with human-generated contours. (A) Histogram of overall subjective quality of auto-contoured influencers, rated from 1-5 where 1 is unacceptable delineation of the organ (i.e., majority of the auto-contour is outside the organ, or majority of the organ is outside the auto-contour), 3 is moderate modifications required to accurately represent the organ (i.e., auto-contour moderately over-contours or under-contours the organ), and 5 is perfect delineation of the organ without modification. (B) Breakdown of subjective quality by organ (top) and treatment progress (bottom). Auto-contoured mandible was found to be worse quality subjectively than the other three, and the auto-contour subjective quality dropped at later radiotherapy fractions. (C) Table of overall objective quality of auto-contoured influencers compared to human-contoured counterpart, assessed with dice similarity coefficients (DSC), mean Hausdorff distances, and 95% Hausdorff distances (HD95%). (D) When grouped by treatment progress, auto-contoured target DSC was found to worsen with treatment progress (p = 0.033). (E) DSC and HD95% for each of the four auto-contoured organs were similar, signifying the auto-contouring algorithm did not perform better for any one organ versus others. (F) When grouped by treatment progress, influencer auto-contour HD95% was found to increase near the end of the treatment. This indicates auto-contour quality worsens at later radiotherapy fractions.

Figure 3 presents the results of subjective and objective comparisons of automated contour with human-adjusted contours created during automated influencer and target contouring steps of the on-line ART treatment workflow. F-numbers from analysis of variance (ANOVA) are shown. Significance of ANOVA tests is denoted with N.S. (not significant) for p > 0.05 or * for p < 0.05 (significant). Figure 3A and Figure 3B show subjective quality of automated influencer contours overall (left), by sites (right top), and by treatment progression (right bottom). Figure 3C to Figure 3F show dice similarity coefficients (DSC) and 95%-percentile Hausdorff distances (HD95%) comparing automated influencer and CTV contours from human contours overall (Figure 3C), arranged by organs (Figure 3E), and arranged by treatment progression (Figure 3D and Figure 3F).

Subjective rating of the automated influencer was overall satisfactory, with 98% scoring at or above 3 and 82% scoring at or above 4. Objectively, automated contours were similar to human-adjusted contours with DSC > 0.93 for influencers and DSC = 0.98 for treatment target CTV on average. The 95-percentile Hausdorff distance (HD95%) for all targets studied was on average 0.81 mm with max 1.87 mm. Similarly, HD95% for automated influencers stayed within 2 mm on average, though for parotids it could be up to 4.83 mm.

Dosimetric impact of adaptation

**Figure 4:**
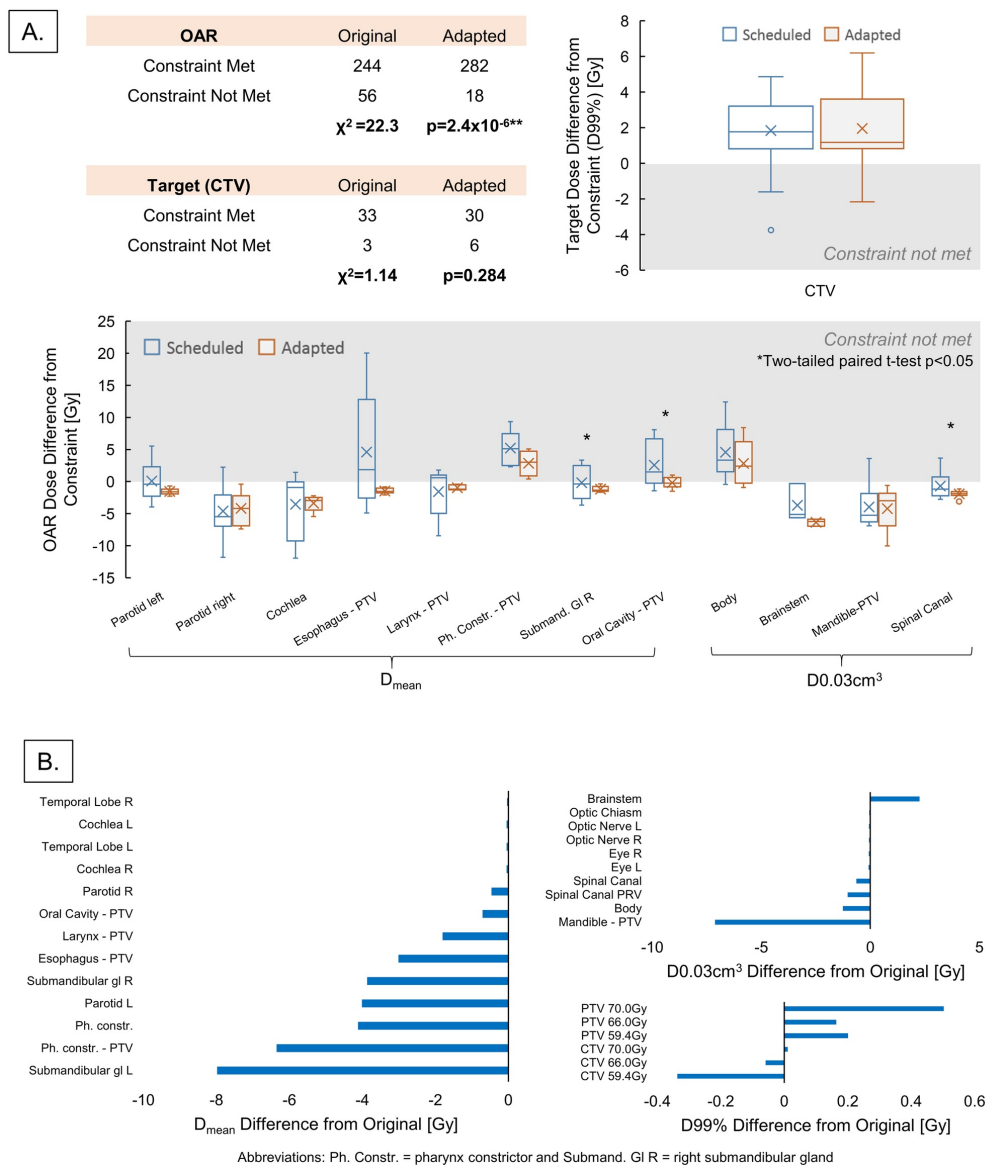
Dosimetric comparison of adapted versus original (scheduled) plans. (A) OAR sparing and target (CTV) coverage for daily adapted plans versus original plan. The table shows the adapted plan met the dose constraints (or clinical goals) for the 300 OARs studied more often than original un-adapted plan (p << 0.001), but not for 36 CTVs studied (p = 0.28). CTV dose difference from the clinical goals is shown on upper right. OAR dose difference from the clinical goals is shown on bottom, grouped by the type of clinical goal (D_mean_ or D0.03cc). (B) Accumulated dose to OARs and targets from one representative adapted plan for a full 70-Gy prescription H&N RT, expressed as a difference from the original plan. The sum of daily doses from adapted plan is generally lower than that from original plan for both D_mean_ and D0.03cc clinical goals (left and top right), but is similar for the CTV D99% with <1 Gy difference for the whole treatment (bottom right). Ph. Constr. = pharynx constrictor; Submand. Gl R = right submandibular gland

Figure 4 compares the adapted plan to the plan in terms of dosimetric parameters. Out of 304 OARs in the five patients being studied, 258 met the clinical goals with the adapted plan versus 234 for the original plan (p = 0.013, two-tailed chi-squared test). Adapted plan met target goals marginally less at 30 versus 33 for the original plan, but this was not statistically significant (p = 0.284). Boxplots corroborate these observations; adapted plans did not reduce dose to target (D99%) versus original plans, but did reduce dose to high-priority OARs (both average dose D_mean_ and hotspot dose D0.03 cm^3^) versus original plans. Here, high-priority OARs are defined as OARs with planned dose >90% of the clinical goals. Oral cavity, pharynx constrictors, and spinal canal consistently benefited from adaptation while other OARs had reduced dose variance overall. Results of paired t-tests comparing adapted and original plan doses are indicated above each OAR (* indicates p < 0.05, ** indicates p < 0.0001). Accumulated doses for one representative adaptive course on Figure 4B also demonstrate adaptation spares most OARs while delivering similar doses to the target compared to the original approved (scheduled) plan, with D99% difference of less than 50 cGy for the whole course.

## Discussion

This paper describes first simulated clinical experiences with a novel semi-automated on-line ART for head and neck cancer based on an O-ring linear accelerator. With a simulated median time of 19 minutes and 34 seconds, the treatment is within a feasible time-frame but would benefit from further algorithm and UI optimization to improve clinical throughput. Automation (influencer / target automated contour and treatment plan generation) only took a median of 6.5 minutes, and the automated contours were both subjectively and objectively satisfactory. Even so, influencer human review consumed a majority of the time at a median of 5 minutes 38 seconds, but varying from as low as 1 minute up to 18 minutes 22 seconds. While moving from a remote, emulated setting to on-site setting may reduce total duration by reducing software lag and alleviating both automation and human contouring times, the fact that human review took longer than automation should hold true. It is additionally noted that the human review time presented here is subject to uncertainty from a variety of factors including but not limited to: (1) possible reduction in influencer review time expected as operators become more experienced with the software and the adaptive process, (2) low-pressure offline simulation may not represent the high-pressure situation expected in on-line adaptation where the patients are on-couch and waiting.

Factors influencing automated contour quality

Satisfactory contour automation was achieved subjectively with 98% of the automated contours scoring higher than 3. Out of the four influencers automatically contoured, the mandible scored subjectively lower than other influencers (more than half <4, p = 0.003). Despite this subjective assessment, there was no statistical evidence that DSC and HD95% among the four automated influencer contours differ (p > 0.05). Qualitative feedback on automated mandible contour cited that the contour failed to delineate high-density bone accurately despite their high visibility on the CT scan, prompting them to adjust the contours. Thus, the current atlas-based automation for mandible may benefit from guidance from intensity-based approach, which can more clearly delineate high-density regions.

In addition, a slight but statistically significant (p = 0.0091) decline in mean automated contour subjective quality was noted over treatment time. Likewise, average influencer HD95% was lower near the end of the treatment (p = 0.021). Target DSC was also observed to decrease with time (p = 0.033), suggesting changes in patient geometry with time, due to tumor volume reduction and weight loss, may negatively affect automated contour quality and thus increase treatment time. In the current workflow, the contour from initial simulation CT is adapted to patient geometry of the day via CT-to-CBCT deformable registration. It is possible the observed decrease in automated contour quality over treatment time can be improved if, instead of simulation CT contours, the daily adapted contours (corrected as needed by human intervention during on-line adaptation) are taken into account.

Dosimetric consequence of adaptation without supervision

**Figure 5:**
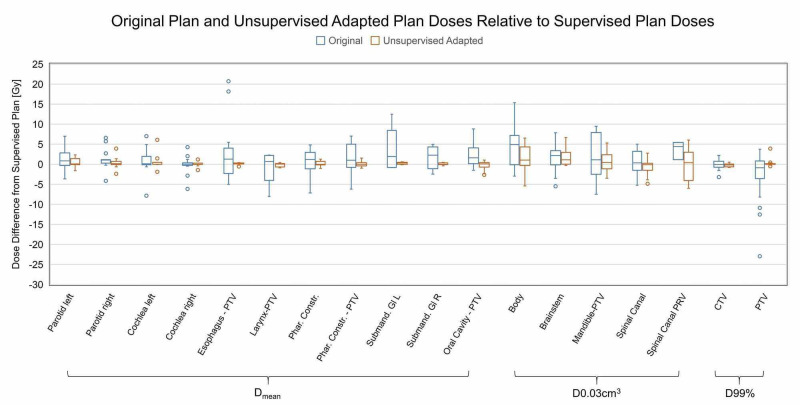
Dose consequences of the lack of supervision on human-adjusted contours (unsupervised adapted) versus not adapting at all (original), compared to supervised plan.

The dosimetric consequences of contour adjustments during on-line ART are explored in Figure 5, which compares dose to target and OAR contours from a “worst-case scenario” plan where human supervision is entirely missing (unsupervised plan) to that from a fully supervised plan. It is important to note that this analysis is NOT approving or advocating using the system unsupervised in any way, but a test to see how the system performs under worst-case scenarios.

All DVH parameters were calculated by applying a plan (e.g., original or unsupervised) to the ground-truth OARs reviewed and approved by the physician. In terms of target doses, the unsupervised plan is nearly identical to the supervised plan, especially compared to the original plan: the unsupervised plan differed -0.2 ± 0.5% from supervised plan for the PTV, while the original plan varied -1.9 ± 6.8% for the PTV. Similarly, OAR doses for the unsupervised plan are more similar to the supervised plan than the original plan, and the original plan frequently overdosed the body contour D0.03 cm^3^ (i.e., global maximum) and oral cavity - PTV D_mean_ compared to supervised plan. Mean doses for human-adjusted Esophagus-PTV, Larynx-PTV, Pharynx Constrictor-PTV, Submandibular Glands, and Oral Cavity-PTV also showed increased dose and variation compared to adapted plan when using the original plan; in contrast, the unsupervised adapted plan showed very similar D_mean_ (<±0.5 Gy for 50% of OARs) to supervised adapted plans. However, the variance in OAR doses is non-negligible especially for D0.03 cm^3^ metrics, up to ±4.2 Gy for brainstem for a 50-70 Gy treatment course. Thus, there is a benefit to ensuring contour outlines influencers and targets as closely as possible. Nevertheless, this analysis showed that even adaptation using auto-generated contours without human review provided improved daily target coverage and OAR sparing consistency compared to treating without adaptation using the original plan. In other words, the uncertainty associated with incorrectly using the system to perform adaptation is still smaller than the uncertainty of treating the entire course with a single original plan.

Strategies to expedite treatment

The current median time of 19.6 min (range 13.2 - 31.3 min) reflects that of remotely accessed emulator, which suffers from operator input lag. Since this product is planned to be an on-site system with minimal lag, actual contour adjustment durations should be slightly shorter than reported. Nevertheless, the times reported here for online adaptive H&N RT is favorable. The median in-room duration for the same five patients treated without on-line adaptation was 7.4 minutes shorter at 12.2 min (range 5.9 - 35.6 min), signifying this workflow is clinically feasible but may risk a loss in patient throughput if any unexpected delays occur. In addition, the 19.6 min online adaptive planning time does not include image acquisition (1.6 min), virtual Mobius-based patient-specific QA procedure (2-3 min), and treatment delivery (1-2 min). Optimization strategies to expedite treatment may further improve this system for efficient clinical use for adaptive H&N RT.

**Figure 6:**
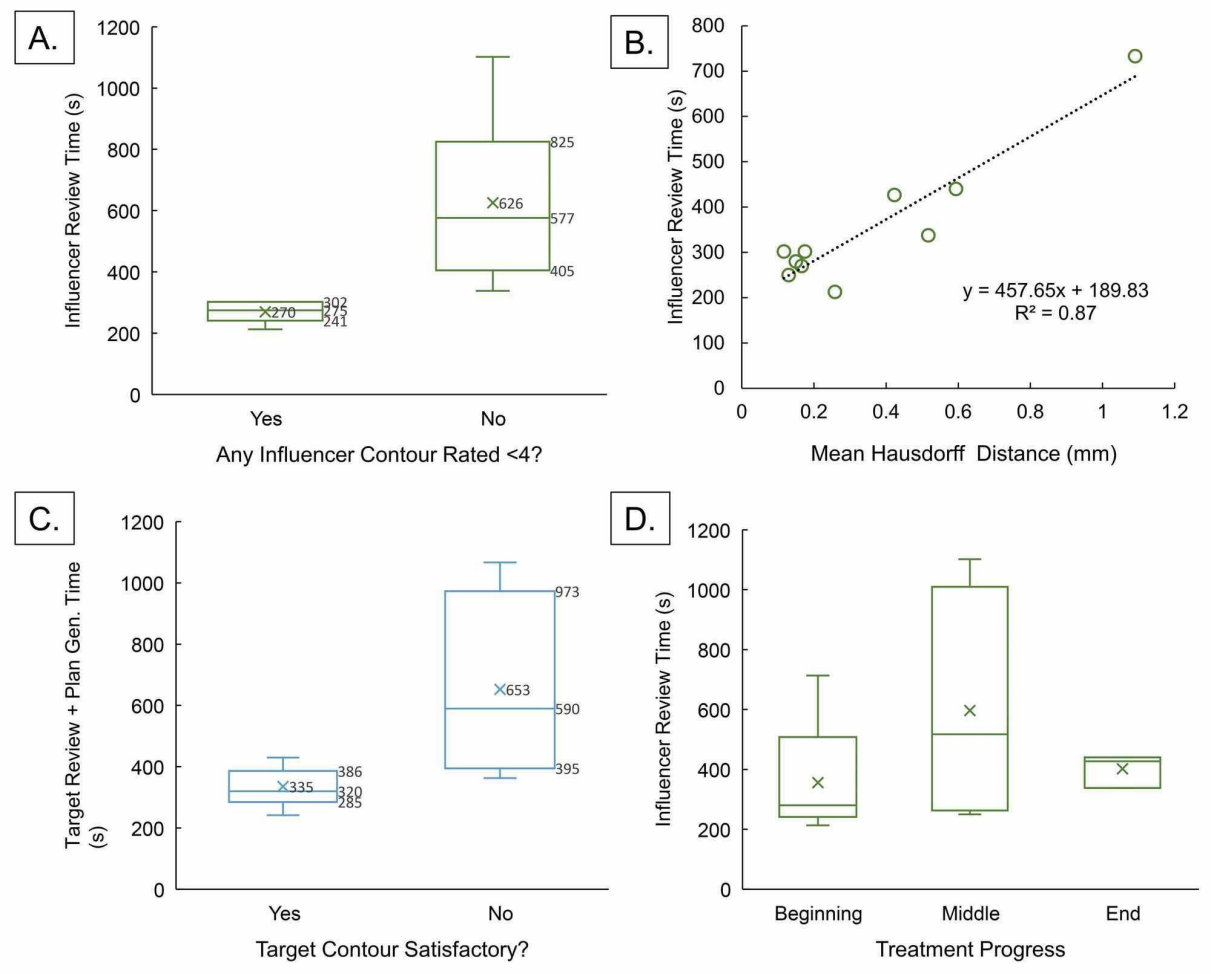
Various factors that significantly impact the duration of human review of automated influencer and target contours during on-line ART. (A) Poor subjective auto-contoured influencer quality extends review time by about 300 seconds. (B) Influencer review time increased linearly with mean Hausdorff distances of the four auto-contoured influencers from their respective human-adjusted contours, indicating worse objective measure of auto-contour quality leads to longer review time. (C) About 360 seconds was saved from target review time if target auto-contour was satisfactory to physicians without adjustments. (D) Treatment progress, which decreased auto-contoured influencer quality in our experience, surprisingly did not affect influencer review time.

Evidence suggests further optimization of automated contour quality may reduce treatment duration by up to 10 minutes (5 minutes during influencer review and 4.5 minutes during target review). Figure 6 identifies various factors that prolonged the duration of on-line ART. Improved accuracy of automated influencers led to a reduction in contour review time to a median of 275s versus 577s, a reduction of ~5 minutes. Likewise, increase in mean Hausdorff distances correlated with influencer review time, increasing review time 458s per 1 mm increase in average Hausdorff distance. Physician re-contour due to poor automated target contours resulted in an increase of median target review time from 320s to 590s (reduction of 4.5 min). This includes extra time from treatment plan re-optimization with approval of a new contour, in addition to time spent on re-contouring itself.

In addition, increased leniency on contour adjustments could expedite treatment with a relatively small penalty on the adapted plan quality. The data on the dosimetric consequence of supervision depicted in Figure 5 suggests OAR D_mean_ from unsupervised plan differs from supervised plan <0.5 Gy on average with a standard deviation <±2 Gy. Assuming normally distributed noise, D_mean_ for all OARs in a 16-fraction treatment with unsupervised treatment course would result in accumulated dose deviating only ±2/√16 = ±0.5 Gy from supervised plan. Again, while no treatment by any means should be entirely unsupervised, a judicious trade-off between leniency in contouring and treatment duration should be considered.

## Conclusions

Feasibility of a novel, semi-automated on-line ART system for various head and neck (H&N) cancer sites was explored in terms of treatment duration, dosimetric benefits, and automated contour accuracy. While the on-line planning workflow duration is clinically feasible, a reduction in patient throughput may occur for a busy clinic. Nevertheless, resulting daily adapted RT plans effectively spare organs-at-risk (OARs) while maintaining a therapeutic dose to clinical target volume (CTV). Automated influencer contours were largely satisfactory both subjectively and objectively, but analyses suggest further improvements in automated contour accuracy may significantly reduce treatment duration. Delineators rated automated mandible contours especially low, citing the need to align better with high-density bones on the CT. A decrease in contour quality (both subjective rating and HD95%) with treatment time was noted as well. At the time of concluding this manuscript, the vendor is working on an improved version (MR1) of this system based on findings in this study. We hope to report evaluation of the improved system soon.
